# First report of rhino-orbital-cerebral mucormycosis after COVID-19 infection in Bulgaria

**DOI:** 10.2217/fmb-2022-0030

**Published:** 2022-07-28

**Authors:** Denis Niyazi, Borislava Toncheva, Tsvetan Tonchev, Deyan Dzhenkov, Kalin Kalchev, Temenuga Stoeva

**Affiliations:** ^1^Laboratory of Clinical Microbiology, University Hospital ‘St. Marina’, Varna, Bulgaria; ^2^Maxillofacial Surgery Clinic, University Hospital ‘St. Marina’, Varna, Bulgaria; ^3^General & clinical pathology clinic, University Hospital ‘St. Marina’, Varna, Bulgaria; ^4^Medical University, Varna, Bulgaria

**Keywords:** Bulgaria, COVID-19, diagnosis, rhino-orbital-cerebral mucormycosis, risk factors

## Abstract

Mucormycosis is a relatively rare infection but with a high mortality rate due to the difficult and time-consuming diagnostic and therapeutic process. The authors present the first case of rhino-orbital-cerebral mucormycosis, histologically and microbiologically proven, in a patient after COVID-19 infection in Bulgaria.

Mucormycosis (zygomycosis, phycomycosis) is an invasive, opportunistic disease caused by saprophytic molds belonging to the order *Mucorales*, class *Zygomycetes* [1]. *Rhizopus*, *Mucor*, *Rhizomucor* and *Lichtheimia* (*Absidia*) species are most commonly associated with human infections [2]. The term ‘mucormycosis’ was introduced by American pathologist RD Baker, and the first case of mucormycosis was described by German pathologist A Paltauf in 1885 [3]. *Rhizopus arrhizus* (previously named *Rhizopus oryzae*) has been reported as the most common causative agent of mucormycosis and is responsible for approximately 70% of all cases [4]. A seasonal variation in the occurrence of this infection is also observed, with most episodes documented in the period from August to November [5]. After candidiasis and aspergillosis, this disease is the third most important fungal infection. Despite its very low incidence, when the disease occurs, it presents with rapid progression and high mortality rates [6,7]. Mortality varies between 30% and 100%, depending on the form and course [7]. After the onset of COVID-19, reports of mucormycosis have increased. Most cases are described in India, and the rhino-orbital-cerebral form is the most common type observed [8–11]. The cases are presented as coinfections with COVID-19 or are diagnosed days to weeks after the viral infection. The authors associate the increased rates of mucormycosis with prolonged corticosteroid treatment in severely ill patients, particularly those with diabetes. As the COVID-19 pandemic progressed and the health crisis worsened, similar reports began to emerge from countries in Europe [12].

The authors present a case of rhino-orbital-cerebral mucormycosis in a patient after COVID-19 infection, hospitalized at the University Hospital in Varna, Bulgaria.

## Case presentation

A 66-year-old male with low socioeconomic status was admitted to the maxillofacial surgery clinic on 26 October 2021 because of a pronounced, painful infiltrate in the right side of the face for 10–15 days. The patient did not report any underlying diseases or risk factors. A month before admission, he was diagnosed with COVID-19 but was not hospitalized. No specific therapy for COVID-19 had been administered. The patient did not receive any immunosuppressive treatment.

**Figure 1.**
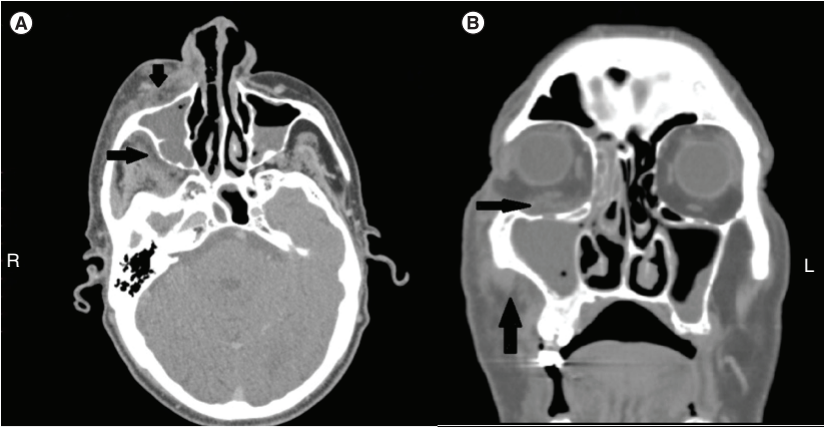
Head CT scan findings. **(A)** Axial head CT scan: necropurulent collection in the infraorbital (horizontal arrow) and infratemporal spaces (vertical arrow). **(B)** Coronal head CT scan: necropurulent collection in the orbital floor (horizontal arrow) and involvement of the buccal space (vertical arrow). Necropurulent collection in the right maxillary and ethmoidal sinuses.

On physical examination, a pronounced infiltrate in the right facial half accompanied by reddened and edematous skin and moderately expressed exophthalmos on the right were documented. No visible pathological changes were observed in the oral cavity. At the time of admission, a blood glucose level of 10.60 mmol/l was found. Additionally, glucose and ketones were detected in the urine sample. During the hospital stay, the blood glucose varied between 7.74 and 16.76 mmol/l. After consultation with an endocrinologist, the oral antidiabetic therapy was adjusted to maintain blood glucose within the normal range. The CT scan detected infiltrates both in the right maxillary sinus and in the orbital floor, accompanied by proptosis of the right eye bulb (Figure 1). On follow-up, lack of adequate contrast in the right internal carotid artery, which was suspicious for thrombosis, was found. The same changes were observed in the cavernous sinus. Osteolysis of the right maxilla was also present. The urgent surgical treatment involved extra- and intraoral incisions in the right facial half. A transpalpebral approach was used to access the periorbital space. The buccal and infratemporal spaces were reached by an intraoral maxillary vestibular incision. After a blunt dissection, dependent drainage was placed. A Caldwell–Luc procedure was used to enter the maxillary sinus and remove the damaged mucosa. Multiple debridement procedures and irrigations with antiseptic agents were performed. In addition to the surgical treatment, adequate management of the hyperglycemia and ketoacidosis, antihypertensive drugs and a combined antimicrobial therapy with amikacin, meropenem and vancomycin were administered.

**Figure 2.**
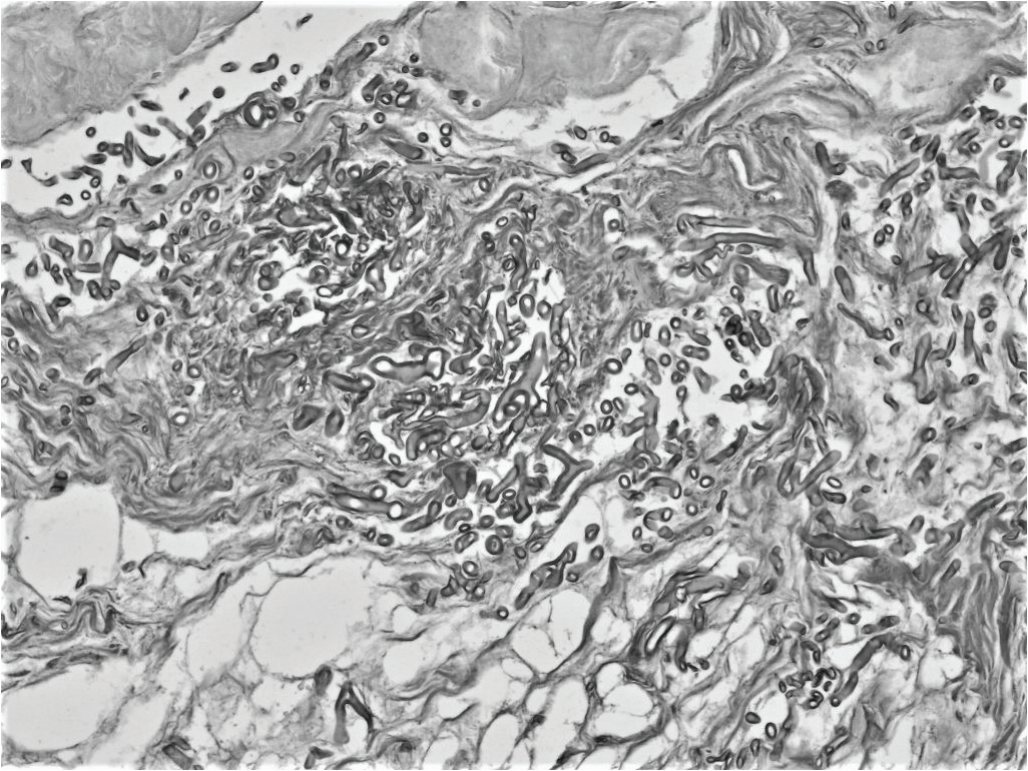
Soft tissues with rough nonseptate hyphae, some branching at right angles (hematoxylin and eosin, ×400).

Histopathological examinations of the affected sinus and soft tissues (hematoxylin and eosin and periodic acid–Schiff staining methods) revealed rough nonseptate hyphae, some branching at right angles; hemorrhages and necroinflammatory debris were seen (Figure 2).

**Figure 3.**
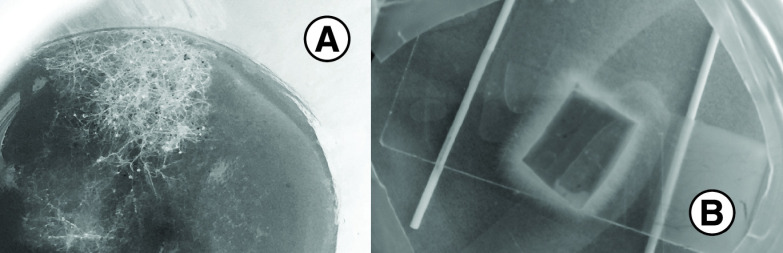
Macroscopic growth of *Rhizopus* spp. **(A)** Visible fungal growth on the surface of the biopsy sample 48 h after sample collection and **(B)** growth of *Rhizopus* spp. on Sabouraud dextrose agar.

Biopsy samples of the necrotic mucosa from the medial border of the sinus were sent for microbiological testing. Rough, nonseptate hyphae, branching at 90°, were observed on direct microscopic examination. Later, when the clinical materials were inoculated on Sabouraud dextrose agar and incubated at 30 °C for 48 h, the growth of white, fluffy mold was observed (Figure 3). Direct microscopic examination of the mold culture demonstrated broad, nonseptate hyphae, some branching at 90° and twisted. Rhizoids and stolons were also documented. Based on the results from the imaging and laboratory examinations, the patient was diagnosed with the rhino-orbital-cerebral form of mucormycosis caused by *Rhizopus* spp. Additionally, *Enterobacter cloacae*, *Candida glabrata* and *Candida zeylanoides* were isolated from the tested clinical samples. After the diagnosis was established, amphotericin B was prescribed to be added to the antimicrobial therapy as an antimold agent. Unfortunately, soon after admission, the patient self-discharged, refusing the necessary surgical and antimicrobial treatment.

A summary of the major clinical symptoms, laboratory results and treatment is presented in Table 1.

**Table 1.** Summary of the main clinical symptoms, laboratory findings, imaging results and treatment in a patient with rhino-orbital-cerebral mucormycosis after COVID-19.

Main clinical symptoms	Blood and urine test results	Histopathological examination (hematoxylin and eosin, periodic acid–Schiff)	Microbiological examination	Imaging study (CT scan)	Treatment
Facial pain Facial edema Reddened and edematous skin Exophthalmos	Hyperglycemia Ketoacidosis	Rough, nonseptate hyphae branching at 90˚ Hemorrhages Necroinflammatory debris	Microscopic examination: rough, nonseptate hyphae branching at 90˚; rhizoids and stolons Culture: white, fluffy mold on Sabouraud agar	Infiltrates in the right maxillary sinus and orbital floor Proptosis of the eye bulb Osteolysis of the maxilla Thrombosis of the cavernous sinus	Surgical treatment Antimicrobial therapy Antidiabetic drugs Antihypertensive drugs

## Discussion

The causative agents of mucormycosis are ubiquitous and found in soil, fruits, vegetables, household dust and organic matter. They can also be detected as colonizers of the upper part of the respiratory system and the distal parts of the digestive tract in healthy individuals [13]. The incidence of mucormycosis varies from country to country. It is between 0.01 and 0.2 cases per 100,000 individuals in Europe, while in the USA the incidence increases to 1.7 per 100,000 individuals annually. In comparison, the incidence of mucormycosis in India is significantly higher, reaching 14 per 100,000 individuals [14,15]. Some authors explain the disease prevalence in India with specific environmental factors such as humid climate and hot weather [16]. In the context of the COVID-19 pandemic, patients with COVID-19 have been diagnosed with fungal coinfections during or after weeks or months of recovery [17]. COVID-19-associated mucormycosis (CAM) has been reported predominantly in India but also in the USA, Austria, France, Italy, Brazil and Egypt [18,19]. Musuuza reports that CAM is responsible for 0.3% of COVID-19 coinfections [20]. Mucormycosis presents in various clinical forms: rhino-orbital-cerebral, pulmonary, cutaneous, gastrointestinal and disseminated [2]. The severe cases with high mortality are due to the ability of hyphae to invade blood vessels, which leads to thrombosis and ischemic infarctions in organs and tissues [21]. Rhino-orbital-cerebral mucormycosis (ROCM) is the most common form observed during the current COVID-19 pandemic, a trend further confirmed by the presented clinical case [22,23]. An acute invasive infection of the nasal cavity, paranasal sinuses and orbit involving facial structures was found in the patient. The symptoms of ROCM may also include eyelid edema, blepharoptosis, internal or external ophthalmoplegia, diplopia and partial or complete loss of vision. Blindness is a major complication that results from an optic nerve infarction due to a central retinal artery or ophthalmic artery occlusion or involvement of the orbital apex. The oral signs of ROCM include palatal necrotic eschars or discoloration, dental pain and loss of teeth [17]. Similar to the reported case, Sen states that the most common symptoms in more than 2800 patients with ROCM associated with COVID-19 are periocular/orbital/facial pain and edema, vision loss, ptosis and proptosis [24]. The presence of necrotic lesions of the hard palate and thrombosis of the cavernous sinus, as seen in the presented clinical case, should be considered an important symptom of ROCM [17].

The predisposing risk factors for developing mucormycosis (including CAM) are varied and include metabolic acidosis (particularly diabetic ketoacidosis), viral-mediated pancreatic damage (COVID-19-associated), prolonged and high-dose corticosteroid therapy, hematological malignancies, neutropenia, advanced age, solid organ or hematopoietic stem cell transplantation, chronic kidney disease, a prolonged intensive care unit stay and the use of deferoxamine, voriconazole and broad-spectrum antimicrobial agents [17,25]. Additionally, COVID-19-associated immunosuppression and pulmonary tissue damage further increase the risk of mucormycosis and other opportunistic fungal infections.

A study from India identifies diabetes as the primary predisposing factor for CAM [26]. Another Indian study reports uncontrolled diabetes to be the most significant risk factor for CAM [27]. In addition, the acute diabetes-like state caused by COVID-19-associated pancreatic damage and the increased glucose levels are also identified as potential risk factors for CAM [17]. The patient from this clinical case did not report any underlying diseases, including diabetes, and it was assumed that the increased and fluctuating blood glucose levels were most probably due to COVID-19-associated pancreatic damage or undiagnosed and untreated diabetes.

In immunocompetent individuals, cell-mediated immunity and the phagocytic cells have a leading role in controlling fungal infections. Hyperglycemia and low pH, associated with diabetic ketoacidosis, negatively affect the phagocytic properties of mono- and polymorphonuclear cells; thus, the intracellular killing of fungal spores is hindered [28]. In addition, *R. oryzae* produces the enzyme ketone reductase, which mediates its ability to flourish at acidic pH and high glucose and ketone levels. Gale demonstrated experimentally that the serum of healthy individuals inhibits the growth of *R. oryzae*, whereas the serum of patients with ketoacidosis stimulates fungal growth [29]. These facts demonstrate that diabetes mellitus further worsens COVID-19-associated immunosuppression. Therefore, maintaining adequate glycemic levels is of prime importance in preventing and controlling COVID-19-associated mucormycosis.

Iron is an important factor in the development and invasion of the Mucorales molds. The low pH decreases the affinity of iron toward transportation proteins (transferrin, ferritin) and the increased concentration of free iron becomes toxic to phagocytic white blood cells, which play the main defense role against the Mucorales molds. The use of deferoxamine in some patients further aggravates the infection, since the chelator acts as a siderophore for the molds and their ability to digest iron increases significantly [30,31]. Hyperferritinemia is one of the prognostic factors in COVID-19 patients, and its persistence is associated with poor outcome. Furthermore, it is a predisposing factor for mucormycosis, leading to rapid fungal growth, phage function alteration, endothelial inflammation and destruction, which decrease the chance of a favorable outcome even more [17].

The presented clinical case demonstrates that the diagnosis of mucormycosis is complex and based on clinical symptoms, histological and microbiological studies and radiological examinations. Although the observed changes are not specific, the imaging studies significantly contribute to the correct diagnosis. In this clinical case, sinus invasion, dislocation and destruction of bone elements were found on CT scan.

The presented clinical case strongly suggests that the microscopy of clinical samples, suspicious for mucormycosis, is an important diagnostic tool. The authors used periodic acid–Schiff, hematoxylin and eosin and Gram methods to confirm the diagnosis. The observation of nonseptate hyphae invading tissues is highly suspicious for mucormycosis. Broad, irregularly branched and nonseptate hyphae were observed. Sometimes they were twisted (ribbonlike) and branching at an angle of 90°. The presence of rhizoids and stolons allowed partial identification of the genus.

Although the representatives of the order Mucorales grow rapidly on solid fungal media, positive cultures are found in only 50% of the cases. Despite the microscopic demonstration of hyphae in the biopsy samples, negative cultures are common. The most likely cause is the fragile nature of the hyphae and their rapid destruction when inoculating the material [15]. The successful isolation of these molds on artificial media, as seen in this case, facilitates their species identification and the testing of their antifungal susceptibility [32].

Treatment of mucormycosis includes several key factors: rapid diagnosis, management of the risk factors, surgical treatment and antimycotic therapy. Early (after achieving stable conditions) and aggressive surgical resection and debridement of the affected tissues is the recommended treatment for mucormycosis [7,17]. The effectiveness of certain antimycotic agents against Mucorales has been demonstrated. Liposomal amphotericin B at a dose of 5 mg/kg/day is the first choice. A higher dose of 10 mg/kg/day is recommended when the central nervous system is involved [15]. In cases of intolerance to amphotericin B, toxicity or lack of effect, posaconazole and isavuconazole can be used as alternatives [15]. If monotherapy is not effective, some authors suggest a combination therapy with lipid polyenes and echincandins/azole (posaconazole, itraconazole) or triple therapy (lipid polyene + echinocandins + azole) [33]. The duration of the systemic antimycotic treatment varies from weeks to months [32]. The Indian Council of Medical Research recommends administering antifungal agents for at least 4–6 weeks [34].

## Conclusion

To the best of our knowledge, this is the first report of COVID-19-associated mucormycosis in Bulgaria. In all individuals who have predisposing risk factors (especially diabetes mellitus) and a history of previous COVID-19 infection, and who also report facial pain, swelling, blurred vision and nasal discharge, COVID-19-associated mucormycosis must be considered as a differential diagnosis, which is a strong argument for diagnostic and therapeutic actions to be taken in due time.

**Executive summary**

- Mucormycosis is a severe infection, often with a lethal outcome, caused by widely distributed molds.
- COVID-19 infection and its complications can be predisposing factors for mucormycosis.
- A complex diagnostic approach that includes various methods (histopathological, microbiological, imaging) is needed to diagnose mucormycosis.
- Adequate management of mucormycosis requires surgical treatment and antifungal therapy.
